# Understanding factors influencing people with disabilities’ participation in sports and cultural activities

**DOI:** 10.1186/s12889-024-17791-9

**Published:** 2024-02-06

**Authors:** Ming Chen, Qianxue Li, Luqi Wang

**Affiliations:** School of Civil Engineering and Architecture, Wuhan University of Technology, No. 122, Luoshi Road, 430070 Wuhan, China

**Keywords:** People with disabilities, Sports and cultural activity, Urban community, Walkability

## Abstract

**Background:**

Improving people with disabilities’ participation in sports and cultural activities benefits their physical and mental health. However, only a few studies have examined the factors that influence participation systematically.

**Methods:**

Using the survey data gathered from 4,319 disabled people living in a district in Wuhan, China, this study explored the impacts of sports and cultural activity participation in terms of individual physiological characteristics, socioeconomic factors, and built environmental features. The sports and cultural facility supply and the walkability index of their community environment were calculated to assess built environment features. Binary logistic regression models were also used to investigate the influence of the aforementioned variables.

**Results:**

There is a significant positive correlation between sports and cultural activity participation and education (OR = 3.44, *p* < 0.01), employment status (OR = 2.04, *p* < 0.01), as well as the number of cultural facilities (OR = 1.33, *p* < 0.01) in the neighborhood area. No significant association was found between the inclination to participate frequently and individual psychological factors.

**Conclusion:**

Regarding people with disabilities’ participation in sports and cultural activities, socioeconomic and built environment factors are more influential than individual psychological ones. The findings can give ideas for identifying targeted and comprehensive interventions to promote a healthy lifestyle for people with disabilities.

## Introduction

Participation in physical and social activities aids in the restoration of people with disabilities’ physical and mental health as well as the improvement of their social cohesion and social support [1–4]. Compared to non-disabled people, disabled people may have a smaller geographic area in which to participate in activities, take longer on average to go out, and face greater spatial and temporal constraints [5–7]. Disabled people usually spend more of their leisure time indoors doing passive activities (e.g., reading, sleeping, or watching television) and less time participating in cultural or outdoor activities [8, 9]. As a result, people living with disabilities are less likely to meet physical activity guidelines and are at higher risk of serious health problems related to inactivity than people without disabilities [10, 11]. They also have a relatively low likelihood of obtaining the psychological and social benefits associated with physical activity [12, 13]. Therefore, to develop more targeted policies and measures, it is important to understand the facilitators and barriers that affect people with disabilities’ participation in sports and cultural activities [14–16].

A variety of factors can influence disabled people’s engagement in physical activities [17]. Gender [18–20], age [18, 21] as well as type and degree of disability [22, 23] are significant influences. The urban built environment, especially neighborhood environment characteristics related to walking, has also been found vital for promoting the general population’s participation in an active lifestyle throughout their lives [24]. On the one hand, several studies have focused on the built environment’s influence on physical activities. For example, Gray et al. found that access to pedestrian lanes, public transportation, parking, recreation, signage, signals as well as public amenities are all important features affecting the walking behavior of people with disabilities [25]. Regarding activity participation, Clarke and George suggested that land use diversity and housing density have a significant impact on the daily activities of people with lower limb disabilities [26]. In terms of community walkability, Schreuer et al. argued that mixed land use, gentler slopes, and greater community connectivity all have a positive influence on people with disabilities’ participation in recreational and cultural activities [27]. On the other hand, there is insufficient research on their sports and cultural activity participation.

Sports and cultural activities are essential components of people’s daily lives. Such activities include singing, dancing, performing instruments as well as playing basketball and football [28]. There are also activities developed for disabled people such as wheelchair basketball. In terms of participation patterns, sports and cultural activities may be more dependent on relevant facilities and social organizations than general physical activities (e.g., walking). Therefore, sports and cultural activity participation can help people of all ages socialize while also improving their physical and mental health as well as their quality of life [29–31]. Studies have found significant differences in sports activity participation between disabled and non-disabled participants [32]. Inadequate facilities, transport, and accessibility issues are common environmental barriers that prevent people with physical disabilities from engaging in sports [11]. However, the majority of prior research has concentrated on access to sports activities. It is essential to acquire a comprehensive understanding of influential factors, such as neighborhood built environment characteristics and socioeconomic factors.

Thus, to create better conditions for people with disabilities’ engagement in cultural and sports activities, this study aims to investigate the influential personal, social, and environmental factors. We assume that the degree of disability, the accessibility of cultural and sports facilities, and walkability of the neighborhood will affect participation in cultural and sports activities. Using survey data gathered from 4,319 disabled people in a district of Wuhan, this paper analyzed the participation rate in cultural and sports activities, accessibility of cultural and sports facilities, and factors affecting participation across three dimensions: individual physiological, socioeconomic, and built environment.

## Methods

### Study area and participants

**Fig. 1:**
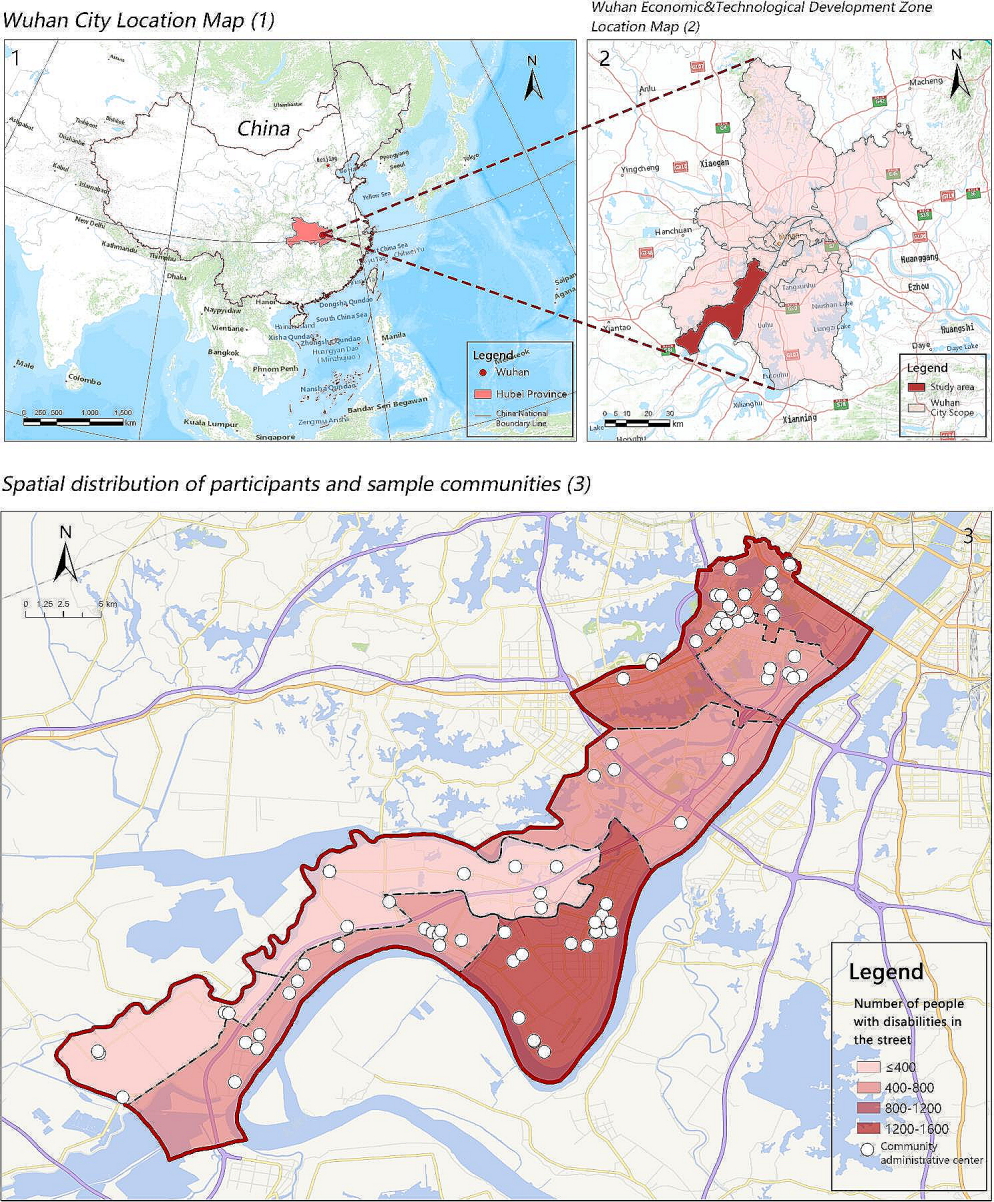
Location of the study area

Located in the southwest of Wuhan, this study’s research area is the Wuhan Economic and Technological Development Zone (WETDZ). It belongs to the urban periphery of Wuhan City (Fig. 1). This area has a total of 86 communities, with seven administrative streets (street districts): Zhuanyang, Zhuankou, Junshan, Shamao, Dunnan, Dongjing, and Xiangkou.

Data from the 2021 National Survey on the Basic Service Status and Demand Information of Persons with Disabilities in WETDZ were used in this study. Initially, the sample size was 5,656. To ensure the accuracy of participation rates, we compared the survey results from 2021 to 2022 and removed six communities where participation rates had changed by 50% or more. This study’s final sample included 4,319 people with disabilities from 80 communities. According to the “Classification and Grading of Disabilities of Persons with Disabilities” (GB/T 26,341 − 2010) implemented by China’s Ministry of Civil Affairs since 2011, severity of disabilities is further divided into four degrees. First-degree represents extremely severe disability, second-degree indicates severe disability, third-degree corresponds to moderate disability, and fourth-degree is considered mild disability. For example, hearing disabilities are classified into four levels: first-degree (hearing loss over 90 dB), indicating profound damage with extreme communication limitations; second-degree (hearing loss between 81 and 90 dB), denoting severe impairment that significantly restricts understanding and communication; third-degree (hearing loss between 61 and 80 dB), representing moderate damage with moderate communication and social participation challenges; and fourth-degree (hearing loss between 41 and 60 dB), reflecting moderate impairment with mild communication and social involvement difficulties.

### Data analysis

#### GIS-based spatial analysis

##### (1) Accessibility of sports and cultural facilities

Accessibility refers to the ease of reaching a location [33]. It can be used to represent the ease with which people with disabilities can access cultural and sports facilities. The cumulative opportunity method [34] was utilized in this research to calculate the number of facilities (resources) within the threshold that are accessible to residents by setting a critical value of travel limit distance (time), with the higher value indicating better accessibility. This is one of the most common accessibility measures employed in planning applications [35]. It is also widely used for job accessibility [36], healthcare [37], recreational parks [38] as well as other services and facilities.

In this study, we measured the accessibility at two levels. At the community level, we calculated the supply of sports and cultural facilities based on the number of such facilities within each community’s 15-minute walking circle. This was determined in ArcGIS using the administrative centers of the sample communities as a starting point and considering the real path time consumption in a traffic isochronous circle. At the district (WETDZ) level, we assessed the number of accessible cultural and sports facilities within 1,000 m and 2,000 m based on road network. This approach helps to illustrate the possible combinations of origin–destination traffic between residential areas and service facilities [39] as well as to map the overall coverage of facilities by the whole network, thereby creating a schematic diagram of the cultural and sports facilities’ accessibility in the WETDZ in a spatially explicit way.

##### (2) Neighborhood walkability

Walkable neighborhoods are characterized by physical attributes that encourage walking, such as connected streets, high residential density, and mixed land use [40]. According to the literature, increased community walkability facilitates physical activity participation [41, 42] and active transportation use [43]. In this study, we used the walkability index to characterize built environment features, including residential density, street connectivity, and land-use mix [44, 45]. Following Sundquist et al. (2011), we used the z-scores method to normalize the three variables in each neighborhood, and the sum of the three z-scores is the walkability index for each neighborhood:

$$ {\text{Walkability}}\,{\text{Index}} = {{\text{z}}_{Populationdensity}} + {z_{Streetconnectivity}} + {z_{LandUseMix}} $$

The neighborhood environment can be defined as a 500-meter-radius circle centered on the participants’ neighborhoods according to existing research [27]. Therefore, the neighborhood walk index for each community in this study was measured based on a 500-meter Euclidean buffer around the community’s administrative center. Population density is calculated by dividing the neighborhood’s area by the number of people within it, while street connectivity is assessed by counting the number of road intersections within a neighborhood. Furthermore, the land-use mix degree is determined based on the entropy measurement, which uses Wuhan City’s POI (Point of Interest) data. Different types of POI are classified based on six functions: industry, housing, green space and square, public facilities, transportation as well as commerce and service industry. Using information entropy [46, 47], the land-use mix is calculated as follows:

$$ \text{H}=-\sum _{x\in U}P\left(x\right)\text{log}P\left(x\right)$$

H represents the entropy of the random variable x, and P(x) is the probability of taking the value of x. When the entropy value is higher, it indicates a greater degree of land-use mix.

#### Statistical analysis

To explore the influential factors of sports and cultural activity participation, we used the binary logistic regression analysis, which is commonly employed when investigating the degree of influence of multiple factors. The dependent variable pertains to whether people with disabilities in the WETDZ regularly participated in cultural and sports activities in the past year (regular participation in cultural and sports activities in the past year = 1, irregular participation in cultural and sports activities in the past year = 0). “Regular participation in cultural and sports activities” means participation in cultural and sports activities 10 times (or more) in the past year. The mathematical expression of the binary regression model is as follows:

$$ \text{ln}(\frac{p}{1-p})=\alpha +{\beta }_{1}{X}_{1}+{\beta }_{2}{X}_{2}+\dots +{\beta }_{n}{X}_{n}$$

where $\text{ln}\left(\frac{p}{1-p}\right)$ represents the probability when the dependent variable takes the value of 1, α is the constant, β_i_ is the regression coefficient, and X_i_ is the respective variable. When the independent variable is changed by one unit (i.e., the average change in Logit p), the regression coefficient β_i_ represents the logarithmic change in the ratio of the probability of occurrence to non-occurrence of the event (i.e., regular participation). The analyses were conducted using the SPSS 26.0 software.

## Results

### Descriptive statistics

Table 1 shows the characteristics of the sample in the WETDZ, including age, gender, education level, disability type and degree, marital status, and type of residence. Based on the sample data, people with disabilities in the WETDZ have the following characteristics:

(1) High degree of aging. The average age was about 50 years old (50.5), with 14.4% over the age of 65.

(2) Low level of education. Only 6.2% of the sample population received tertiary education or higher, which was significantly lower than the percentage for people without disabilities.

(3) The physical disability group accounted for the largest proportion, with most having a second- or third-degree disability. In terms of severity, the sample population in the WETDZ mainly had second- and third-degree disabilities, accounting for 70.6% of the total.

(4) Employment level was relatively low. Only 30.1% of the sample population was employed.

(5) The married population predominated. The majority of people with disability were married (66.8%).

**Table 1:** Profile of the sample population in the WETDZ

Variable	Category	N	Percentage (%)
Gender	Male	2,514	58.2
	Female	1,805	41.8
Education level	Primary school and below	878	20.3
	Middle School and High School	3,176	73.5
	College and higher	265	6.2
Type of disability	Visual disability	389	9.0
	Physical disability	2,073	48.0
	Deaf-mute	427	10.1
	Intellectual disability	348	8.0
	Mental disability	940	22.0
	Multiple disabilities	142	2.9
Degree of disability	First-degree (extremely severe)	573	13.3
	Second-degree (severe)	1,656	38.3
	Third-degree (moderate)	1,393	32.3
	Fourth-degree (mild)	697	16.1
Employment status	Unemployed	1,688	39.1
	Employed	1,302	30.1
	Other	1,329	30.8
Marital Status	Single	955	22.1
	Married	2,883	66.8
	Divorced	194	4.5
	Widowed	151	3.5
	Other	136	3.1
Type of residence	Rental housing	183	4.2
	Affordable housing	208	4.8
	Self-built housing	1,130	26.2
	Commercial housing	1,607	37.2
	Other housing	1,191	27.6

**Fig. 2:**
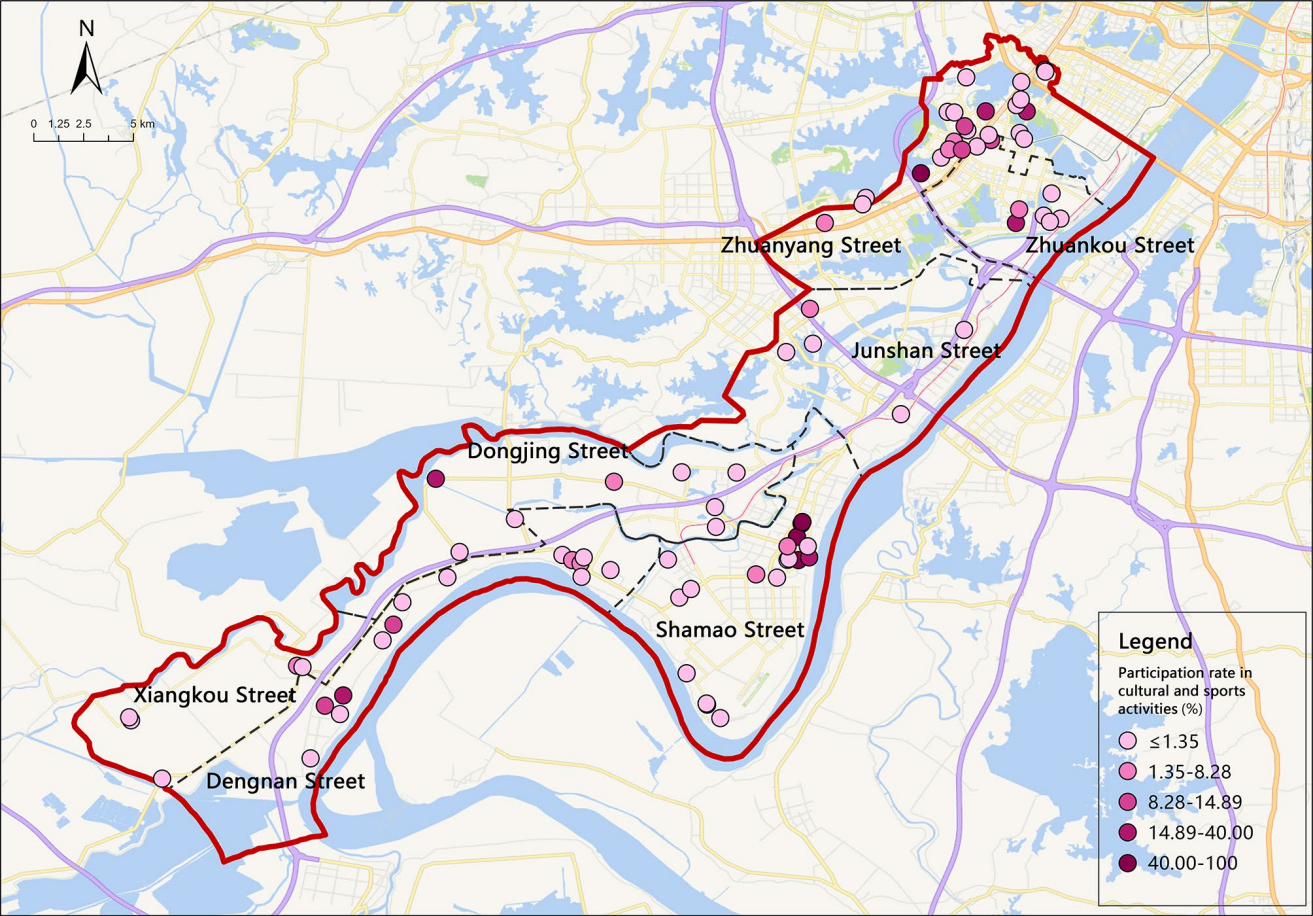
Spatial distribution of participation rates in sports and cultural activities in the WETDZ

An analysis of the distribution of people with disabilities in each street in the WETDZ showed that Shamao Street had the highest total number of disabled people at 1,339. To depict the participation status (Fig. 2), the percentage of those with disabilities who often participate in cultural and sports activities in each community was calculated and divided into five levels. In the WETDZ, 7.57% of people with disabilities regularly participated in community cultural and sports activities, which was considerably lower than the national average participation rate in 2021 (23.7%). The spatial distribution map below shows that participation in community sports and cultural activities was generally high in Zhuanyang, Dengnan, and Shamao streets but generally low in Xiangkou and Junshan streets. Also, the distribution of high-participation communities was relatively clustered in Zhuankou, Zhuanyang, and Shamao streets, but relatively scattered in Dengnan and Dongjing streets.

**Fig. 3:**
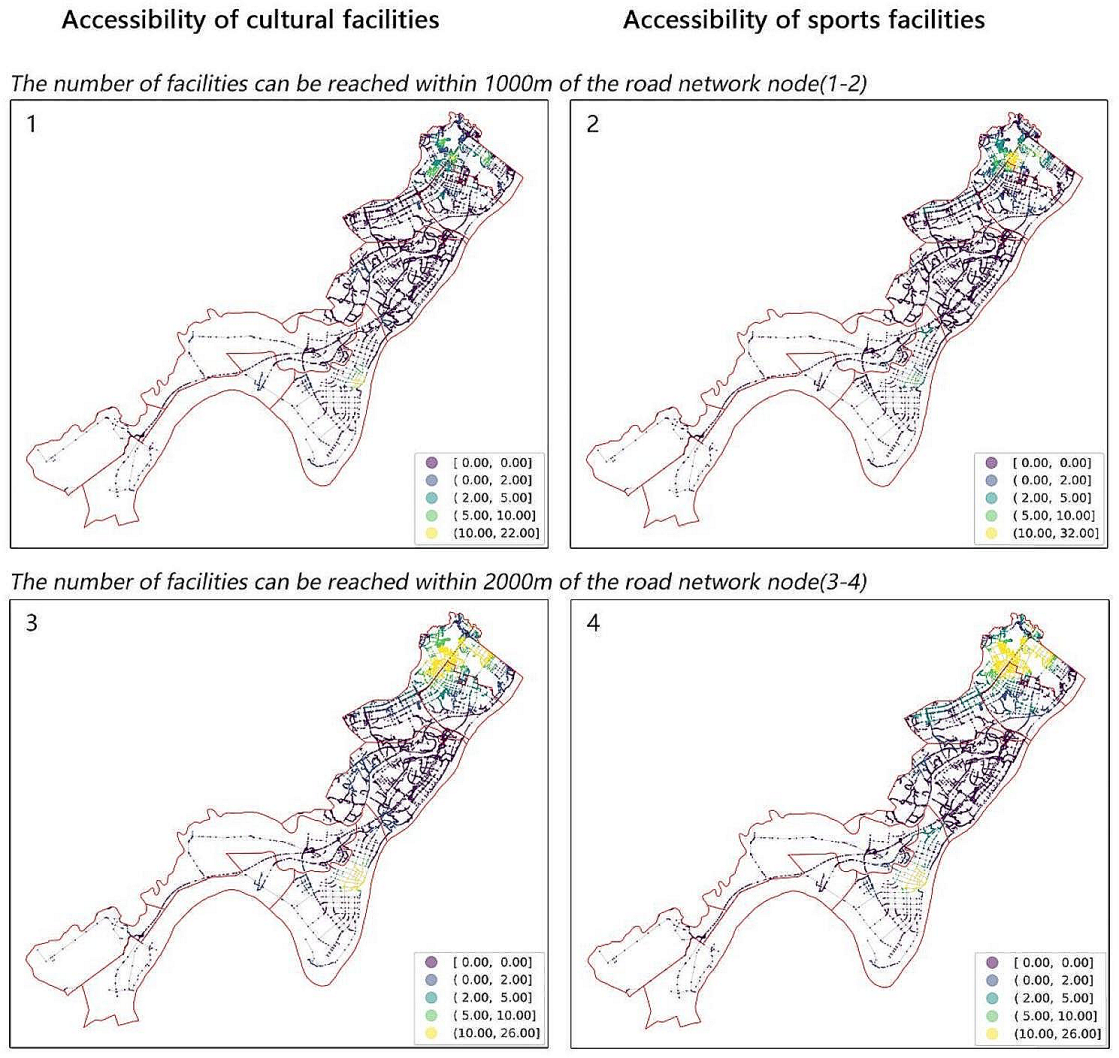
Accessibility of cultural and sports facilities in the WETDZ

The accessibility of cultural and sports facilities in the WETDZ was derived by constructing a traffic network dataset through GIS and calculating the number of accessible cultural and sports facilities within 1,000-meter and 2,000-meter buffer areas of each road node, respectively (Fig. 3). If the number of facilities within the critical value reached ten or higher (shown in yellow), the accessibility was considered high. The results showed that cultural facilities were most accessible in Zhuanyang and Shamao Streets, with the former having a more balanced distribution and the latter having a concentrated one. Moreover, the accessibility of sports facilities was highest in Zhuanyang and Zhuankou Streets, with both having a clustered distribution. In terms of the number of facilities and road accessibility, the cultural and sports facilities’ accessibility in Zhuanyang, Zhuankou, and Shamao streets was good, while it was poor in Dongjing, Xiangkou, Junshan, and Dengnan streets.

When using the 2,000-meter range of road network nodes as the critical value, the accessibility of facilities increased substantially in Zhuankou and Zhuanyang streets, increased slightly in Shamao Street, and changed minimally in the remaining streets. Overall, cultural and sports facilities were more accessible in Zhuanyang, Zhuankou, and Shamao streets than in other areas.

### Factors influencing participation rate in cultural and sports activities

Regression models (Table 2) were created to analyze the effects of participation in cultural and sports activities among people with disabilities in the WETDZ. Three models were constructed based on three dimensions: individual physiological, socioeconomic, and built environment. The level of participation was used as the dependent variable, with frequent participation in cultural and sports activities in the past year = 1 and infrequent participation = 0.

Regarding the models’ global tests, the findings of the Omnibus Tests of Model Coefficients showed that the significance level of all three models was less than 0.05, indicating they were significant overall. The goodness-of-fit test (Hosmer–Lemesho test) was also performed on all three models, and the results were all greater than 0.05, signifying that the model fit was good.

**Table 2:** Logistic regression results of factors influencing the participation rate of people with disabilities in cultural and sports activities

	Model 1: Individual physiological variables	Model 2: Model 1 + socioeconomic variables	Model 3: Model 2 + built environment variables
Variable	OR 95% CI	OR 95% CI	OR 95% CI
Gender			
Female	1.00	1.00	1.00
Male	1.06 (0.84–1.34)	0.88 (0.68–1.13)	0.84 (0.63–1.11)
Age	1.00 (0.99–1.01)	1.01 (0.99–1.03)	1.01 (0.99–1.03)
Type of Disability			
Visual	1.00	1.00	1.00
Physical	1.30 (0.83–2.04)	1.31 (0.83–2.07)	1.05 (0.64–1.72)
Deaf–Mute	1.57 (0.91–2.69)	1.38 (0.79–2.40)	1.08 (0.59–1.99)
Intellectual	0.70 (0.36–1.37)	1.16 (0.57–2.34)	0.91 (0.43–1.94)
Mental	1.08 (0.64–1.82)	1.52 (0.89–2.60)	1.36 (0.76–2.44)
Multiple	0.52 (0.19–1.39)	0.73 (0.27–2.01)	0.48 (0.16–1.44)
Degree of disability			
First-degree	1.00	1.00	1.00
Second-degree	1.01 (0.66–1.54)	0.83 (0.54–1.27)	0.87 (0.54–1.41)
Third-degree	1.34 (0.88–2.04)	0.96 (0.63–1.49)	1.19 (0.74–1.92)
Fourth-degree	1.35 (0.85–2.14)	0.87 (0.54–1.40)	1.21 (0.71–2.05)
Education level			
Primary school and below		1.00	1.00
Middle School and High School		1.43*(0.95–2.16)	1.16 (0.75–1.81)
College and higher		1.93**(1.11–3.35)	3.44***(1.86–6.36)
Employment status			
Unemployed		1.00	1.00
Employed		2.19***(1.59–3.01)	2.04***(1.44–2.90)
Other		1.17 (0.77–1.76)	1.31 (0.84–2.06)
Marital Status			
Single		1.00	1.00
Married		0.82 (0.56–1.21)	0.87 (0.56–1.34)
Divorced		0.31***(0.13–0.75)	0.47 (0.19–1.19)
Widowed		0.47*(0.20–1.11)	0.46*(0.19–1.14)
Other		1.63 (0.61–4.31)	2.65*(0.93–7.53)
Type of Residence			
Rental housing		1.00	1.00
Affordable housing		0.93 (0.37–2.40)	1.39 (0.54–3.63)
Self-built housing		1.38 (0.68–2.82)	0.70 (0.33–1.47)
Commercial housing		2.33**(1.16–4.67)	0.67 (0.32–1.42)
Other housing		0.39**(0.18–0.85)	0.34***(0.15–0.77)
Cultural facility supply			1.33***(1.28–1.38)
Sports facility supply			0.78***(0.72–0.85)
Walkability			0.92 (0.83–1.03)
Model Chi-Square	22.072**	183.959***	571.057***
-2 log likelihood	2294.376a	2,132.489a	1,745.391a
Nagelkerke r^2^	0.012	0.100	0.298

Note: *p<0.1,**p<0.05,***p<0.01; Dependent variable: 0 = infrequent participation in cultural and sports activities, 1 = frequent participation in cultural and sports activities

Model 1 (individual physiological model) only contains respondents’ age, gender, and type and degree of disability. The results revealed that there was no significant association between the inclination of individuals with disabilities to participate frequently and their gender, age, and degree and type of disability.

Model 2 (socioeconomic model) included both individual physical and socioeconomic factors. Education, employment, marital status, and housing type were found to have significant effects. People with disabilities who were well-educated, employed, and lived in commercial housing were relatively more likely to engage in cultural and sports activities, while those who were divorced or widowed and reside in other housing types were relatively less likely to participate. Specifically, the employed were 119% more likely to join in sports and cultural activities than the unemployed (*p* < 0.01). In terms of marital status, divorced and widowed individuals were 69% and 53% less likely to participate in sports and cultural activities, respectively, than unmarried people. Concerning housing type, disabled people who lived in commercial housing were 2.3 times more likely to engage in cultural and sports activities than those who reside in rental housing; moreover, individuals whose housing type was unclear were 61% less likely to participate (*p* < 0.05).

Model 3 expanded on Model 2 by adding the built environment variables. The results showed that education level, employment status, marital status, housing type, and adequate cultural and sports facility supply all had significant effects on people with disabilities’ engagement in cultural and sports activities. Education-wise, participation in cultural and sports activities was significantly more likely for those with a college degree or higher (OR = 3.44, *p* < 0.01) than those with an elementary or lower level of education. Moreover, the engagement rate in cultural and sports activities was 104% higher among employed disabled people. Furthermore, being married (OR = 2.65, *p* < 0.1) had a significant positive effect, while being widowed (OR = 0.46, *p* < 0.1) had a significant negative impact on participation in comparison to being single. As for housing type, living in other housing (e.g., borrowed or temporary housing) had a significant negative effect (OR = 0.34, *p* < 0.01) on participation in comparison to residing in rental housing (OR = 0.34, *p* < 0.01). Additionally, the availability of cultural facilities had a significant positive effect, with each additional cultural facility increasing the likelihood of engagement in cultural and sports activities by 33%. Surprisingly, the number of sports facilities had a significant negative effect, as each additional sports facility decreased the probability of frequent participation by 22%.

## Discussion

This study explored factors affecting people with disabilities’ participation in sports and cultural activities in terms of physiological, socioeconomic, and built environment characteristics. Regarding individual physiological factors, we discovered that age, gender as well as type and degree of disability had limited influence on participation in cultural and sports activities. This finding, however, is inconsistent with our hypothesis and previous research. Several studies [19, 20, 48] have found that gender plays an important and influential role in people with disabilities’ physical activity, with women reporting lower levels of physical activity than men. Age also affects their physical activity, with older people often being less active than younger individuals [18, 21]. Physical impairment and low self-esteem were also found to have an impact on their involvement in sports activities [49]. In terms of educational attainment, our results highlighted that it had a significant positive effect on participation rate. This is in line with a study conducted in Poland [50], which found education level to be significantly correlated with engagement in tourism activities by older adults with disability. A lack of education may affect how individuals with disabilities perceive the value of cultural and physical activities.

As for socioeconomic factors, our study confirmed the importance of employment status. Previous research has highlighted that cost is a critical concern for people with disabilities who want to travel or take part in a variety of activities [51–53]. Being employed can contribute to a stable income, thus facilitating sports and cultural activity participation. We also found that marital status, particularly divorce and widowhood, had a significant negative effect on physical and cultural activity engagement. This finding echoes existing research indicating that family support increased the level of physical activity among people with intellectual disabilities [54]. Having social or supportive relationships was also positively associated with better health and well-being [55]. Individuals with disabilities who were well-cared for by their families were more likely to communicate with others and participate in social activities. In addition, they often preferred to be accompanied by close family members or close friends on their trips.

Concerning built environment factors, it was found that while the number of cultural facilities had a significant positive effect on the participation rate, the availability of sports facilities had a significant negative impact. Most of the existing cultural facilities in the WETDZ are public welfare facilities, i.e., the government and society are the main operating entities, providing free or low-cost services to residents. Moreover, sports facilities are mostly market-based, with operators charging residents for their services. Given the cost issue, most disabled people in the WETDZ may rely on cultural facilities. In addition, proximity does not necessarily guarantee higher participation rates. People with disabilities may be hesitant to participate in sports at the nearest facility due to concerns about being observed by their neighbors while participating in group activities. The result regarding neighborhood walkability is also inconsistent with our assumption. Although many scholars [27, 56] have underscored that neighborhood walkability promotes physical activity, we discovered that it was not related to sports and cultural activity participation. People with disabilities may prefer to travel by private car [44, 57]. In our study area, 26 of 80 communities lack public sports and cultural facilities within a 15-minute walking distance, which may limit the chances of disabled people walking to spaces holding cultural and sports activities and engaging in them.

Based on our findings, since participating in sports and cultural activities provide numerous benefits for people with disabilities, comprehensive efforts should be taken to promote involvement. First, the equality of access to sports and cultural activities must be improved. A sufficient quantity of sports and cultural facilities is vital for both disabled and non-disabled people. Since many individuals with disabilities are socioeconomically disadvantaged, more sports facilities should be made available for them at a lower price. A greater number of existing facilities can also be modified using inclusive design concepts [58]. Second, awareness of the benefits of sports and cultural activities must be raised. Many respondents in our study lacked a high level of education. They may be unaware of the advantages of outdoor sports and cultural activities. In a random-sample interview in our study area, several respondents and their family members mentioned that they do not want to travel beyond their immediate neighborhood. They also stated that their usual pastime is listening to the radio and music at home and that they do not want to go out. People are sometimes unaware of nearby sports or cultural facilities that are accessible to them [59]. While many disabled people have a lot of free time [51], it is critical to encourage them to take part in sports and cultural activities for their physical and mental health. Third, based on the collaboration of sports and cultural groups and disability organizations, targeted strategies to increase participation should be developed. The appropriate design and adaptation of sports and cultural programs, tailored to the specific type and degree of disability, should be explored. A structured exercise program can encourage individuals with disabilities to go out and engage in more social and intense daily pursuits. Additionally, improving access to the adaptive sports programs could facilitate sustained participation [60]. This endeavor requires the support of professionals working in disability sports associations and the community [61].

## Conclusions

People with disabilities may encounter many barriers when it comes to participating in cultural and sporting activities as well as integrating into society. Based on a survey of 4,319 disabled people from 80 communities, this study contributes to our understanding of the factors influencing participation in sports and cultural activities among disabled people. We found that socioeconomic and built environment factors are more influential than individual psychological variables. The results also highlighted the significance of education level, employment status, and the availability of cultural facilities in the surrounding area. The findings can give ideas for identifying targeted and comprehensive interventions to promote a healthy lifestyle for people with disabilities.

Although we examined a variety of factors, such as the number of facilities and walkability in the neighborhood area, we did not include the detailed accessible environment (e.g., signs, road quality, slope, handrails), which might have a significant impact on disabled people’s willingness to travel. For people with disabilities, particularly those who use wheelchairs or are blind, access to barrier-free bus lines and metro stations is crucial. More research is needed to explore the relation between access to barrier-free public transport and activity participation. Future studies should aim for a more comprehensive examination of the built environment factors related to inclusive design concepts, and investigate the preferences of people with disabilities in terms of sports and cultural activities.

## Data Availability

The datasets generated and/or analyzed during the current study are not publicly available due to consideration of participants’ privacy, but are available from the corresponding author on reasonable request.

## Abbreviations

WETDZ: Wuhan Economic and Technological Development Zone
POI: Point of Interest
